# Artificial intelligence will change the research environment in dental medicine dramatically: will algorithms replace literature reviews in the near future?

**DOI:** 10.1093/dmfr/twaf041

**Published:** 2025-05-12

**Authors:** Michael M Bornstein, Yannick M Staedler, Philippe C Cattin

**Affiliations:** Department of Oral Health & Medicine, University Center for Dental Medicine Basel UZB, University of Basel, Basel, CH-4058, Switzerland; University Library of Basel, University of Basel, Basel, CH-4056, Switzerland; Department of Biomedical Engineering, Faculty of Medicine, University of Basel, Allschwil, CH-4123, Switzerland

Since a decade, artificial intelligence (AI) has become increasingly popular and relevant in the field of dentomaxillofacial radiology; initially confined to research, AI-assisted diagnostic tools are now increasingly integrated into routine dental practice.^1^^,^^2^ Over just the last 5 years, AI has emerged as one of the most relevant topics in submissions to *Dentomaxillofacial Radiology* (*DMFR*), with an internal 2024 analysis revealing that 60% of the top-cited articles over the previous half-decade were AI-related.^3^ This dramatically emphasizes the relevance of this topic for our specialty—and we are constantly working as a relevant journal in this field to remain at the forefront of this AI revolution in dental medicine.

Only a few years ago, AI-driven large language models (LLMs), such as OpenAI’s GPT series, Google’s Gemini series, and DeepSeek, began demonstrating their capacity to emulate human intelligence across a broad spectrum of tasks by leveraging vast neural network architectures and extensive training corpora. These models engage in fluent, context-aware dialogue and writing, including the composition of coherent texts and conversations that convincingly replicate human emotions such as humor and sarcasm. Recently, OpenAI’s GPT-4.5 was officially reported to have passed the Turing test.^4^ In this imitation game used to gauge machine intelligence, ChatGPT-4.5 was judged to be indistinguishable from a human 73% of the time by a group of 126 undergraduate psychology students.^5^

Google’s Co-Scientist (based on Gemini 2), unveiled in 2025, adopts a multi-agent approach. It uses specialized sub-agents to generate, rank, critique, and iteratively refine hypotheses, as well as to perform web searches in pursuit of research goals.^6^ Such an approach is also reflected in Gemini 2.5 Pro, which integrates advanced reasoning capabilities and can deploy multiple specialized agents before generating responses. In certain implementations, Gemini even has live web access, enabling real-time information retrieval.^7^ DeepSeek, an open-weight LLM from China, has rapidly established itself as a top-tier model for academic tasks by combining reasoning capabilities, efficient training, and practical tools for literature synthesis, manuscript editing, and hypothesis generation.^8^

Despite their performance in imitating human language, LLMs are prone to hallucinations, a phenomenon in which the model generates information that sounds plausible but is factually incorrect or unsupported. This issue persists even when LLMs are trained on high-quality scientific literature (eg BioGPT).^9^ To mitigate this problem, Retrieval-Augmented Generation (RAG) has emerged as a solution. Retrieval-Augmented Generation operates through a 2-stage process: retrieval and generation.^10^ In the *retrieval* phase, the system searches a domain-specific database to identify documents relevant to a user’s query (eg abstracts from Semantic Scholar). During *generation*, an LLM (eg ChatGPT 4) synthesizes a response by integrating its internal knowledge with the retrieved content, ensuring that the output is both coherent and contextually grounded. Retrieval-Augmented Generation systems significantly reduce the likelihood of hallucination by grounding responses in verifiable sources. Furthermore, because RAG systems dynamically access external databases, they can incorporate the most recent research findings without requiring model retraining, ensuring that responses reflect current knowledge. By combining the generative fluency of LLMs with the factual precision of information retrieval, RAG represents a significant advancement in the development of trustworthy and domain-adaptable AI systems for scientific and clinical applications. Currently, several RAG-based tools (eg Elicit, Consensus, Scite.ai, Semantic Scholar, Web of Science AI, Scopus AI) are already available to help researchers rapidly generate literature overviews and drafts, provided they are used with proper priming and prompting.

Large language models are therefore on the verge of displaying disruptive potential in scientific writing, where traditionally publications have been grouped into (1) classical research articles presenting data from clinical trials or experimental set-ups, and (2) literature reviews that, in the form of narrative reviews or systematic reviews, do not include original data. It is important to underline here that high-quality literature reviews, authored by leading experts in their field, currently count amongst the most read and highly cited articles for the scholarly journals in which they are published and remain, to date, an important article type. However, classical research articles and literature reviews have been historically conceived, written, and revised by humans—a fact that now seems to be changing with dramatic speed.

In contrast to classical narrative reviews that are at risk to rely and include somewhat outdated articles and might miss the most recent publications once they have passed the review process and are published, AI-generated narrative reviews can include the latest evidence available. This is basically only one mouse click away, that is, dramatically speeding up the availability of up-to-date information. Furthermore, the literature search can be repeated whenever one feels the need for an update. One could even speculate that in the future, the quality of narrative reviews may be more related to the LLM and the prompt used than the expert who historically was at the center of writing such a mansucript.^11^ Will experts simply be needed for a qualitative and factual assessment of the written text and the articles included? This in turn also raises the question on the scientific value of and need for such classical narrative reviews in the future, when it will be very likely possible to generate such a manuscript over a couple of hours by almost anybody capable of priming and prompting with the assistance of a dedicated LLM instead of weeks and months by one or more specialists in the field.

When it comes to systematic reviews, which are considered the gold standard for evidence-based decision making for diagnosis, therapy planning, and follow-up in medicine and dentistry, LLMs have recently shown to hold great potential to reduce the significant costs and time needed for the involved authors.^12^ This recent study has demonstrated that costs involved can be reduced by almost 90%, and the AI-driven algorithms can shorten the time required to screen potential articles from months to a single day or less. For the future, the authors predict that LLMs might facilitate and assist also with the data extraction process, another crucial and time-consuming part of systematic reviews.

The arguments discussed above clearly highlight that the use and impact of AI, especially LLMs, in dental research is a relevant issue with disruptive potential. Scientific journals in the field of dentistry need to highlight the opportunities and also risks that come with an increased use of AI-assisted research and should offer recommendations and guardrails on its proper use and handling. These should be coming soon, as we need to be proactive in such a dynamic environment. On these lines, the British Institute of Radiology (BIR) has recently offered guidance on this pressing issue for their journals, which include *DMFR*. These guidelines also offer recommendations for the 2 following topics: (1) AI and authorship: authorship is limited to those who have made a significant contribution to the design and execution of the work to be published. Any contributors whose participation does not meet the criteria for authorship should be acknowledged but not listed as authors. The BIR journals, published in partnership with Oxford University Press (OUP), follow OUP’s policy on AI and authorship. Natural language processing tools driven by AI do not qualify as authors. Artificial intelligence tools cannot meet the requirements for authorship as they cannot take responsibility for the submitted work. (2) Artificial intelligence and manuscript preparation: AI tools can be used for writing manuscripts or language editing as long as they are openly declared. In practice, authors using AI tools in the writing of a manuscript, production of images or graphical elements of the paper, or in the collection and analysis of data, must be transparent in disclosing this in the cover letters to editors and in the “Materials and Methods” or “Acknowledgements” section of the respective manuscripts. Editors and peer reviewers are and need to be on the lookout for nondisclosed or inappropriate use of AI within submitted manuscripts.

We feel that AI is an incredibly fast-developing field, and many innovations in this context that feel groundbreaking today might already be outdated or updated and improved after a short timeframe, sometimes only after months, and not years. This has even become more evident since LLMs were introduced widely to the public, not even 5 years ago. These AI-driven tools are now on the verge of changing our academic work and also our means of communication—for example, scientific publications—probably forever. It might be that some of the classical forms of these publications, such as narrative or systematic reviews, will soon cease to exist in their current form and may evolve into AI-assisted, living documents that are continuously updated and verified. This again underlines our responsibility to stay abreast of these developments and try to lead and guide the way for our scientific community.
